# A model of environmental limitations on production of *Agave americana* L. grown as a biofuel crop in semi-arid regions

**DOI:** 10.1093/jxb/ery383

**Published:** 2018-12-31

**Authors:** Nicholas A Niechayev, Alexander M Jones, David M Rosenthal, Sarah C Davis

**Affiliations:** 1 Voinovich School of Leadership and Public Affairs, Ohio University, Athens, OH, USA; 2 Department of Environmental and Plant Biology, Ohio University, Athens, OH, USA; 3 University of Essex, UK

**Keywords:** CAM, crassulacean acid metabolism, drought tolerance, bioethanol, temperature response, desert crops, bioenergy, light intensity

## Abstract

Plants that use crassulacean acid metabolism (CAM) have the potential to meet growing agricultural resource demands using land that is considered unsuitable for many common crop species. *Agave americana* L., an obligate CAM plant, has potential as an advanced biofuel crop in water-limited regions, and has greater cold tolerance than other high-yielding CAM species, but physiological tolerances have not been completely resolved. We developed a model to estimate the growth responses of *A. americana* to water input, temperature, and photosynthetically active radiation (PAR). The photosynthetic response to PAR was determined experimentally by measuring the integrated leaf gas exchange over 24 h after acclimation to six light levels. Maximum CO_2_ fixation rates were observed at a PAR intensity of 1250 µmol photons m^–2^ s^–1^. Growth responses of *A. americana* to water and temperature were also determined, and a monthly environmental productivity index (EPI) was derived that can be used to predict biomass growth. The EPI was calculated as the product of water, temperature, and light indices estimated for conditions at a site in Maricopa (Arizona), and compared with measured biomass at the same site (where the first field trial of *A. americana* as a crop was completed). The monthly EPI summed over the lifetime of multi-year crops was highly correlated with the average measured biomass of healthy 2- and 3-year-old plants grown in the field. The resulting relationship between EPI and biomass provides a simple model for estimating the production of *A. americana* at a monthly time step according to light, temperature, and precipitation inputs, and is a useful tool for projecting the potential geographic range of this obligate CAM species in future climatic conditions.

## Teaser

Physiological responses to variation in photosynthetically active radiation, temperature, and water can be used to predict the growth of *Agave americana* L. in field conditions.

## Introduction

Of the three major photosynthetic pathways, crassulacean acid metabolism (CAM) has substantial advantages in semi-arid and xeric regions (Davis *et al.*, 2014). High-yielding CAM species (e.g. *Agave* and *Opuntia*) have approximately four times greater water use efficiency (WUE) than agricultural crop species that use C_4_ photosynthesis, and about six times greater WUE than agricultural C_3_ photosynthetic species (Borland *et al*., 2009, 2014; Davis *et al.*, 2014; X. Yang *et al.*, 2015). Despite the common perception that CAM species are low yielding, commercial *Agave* crops have annual biomass productivities ranging from 8.5 Mg ha^–1^ year^–1^ to 22 Mg ha^–1^ year^–1^ (Davis *et al.* 2014) and may have even greater potential productivities (Davis *et al*., 2010, 2014, 2016). For comparison, productivity of C_4_ photosynthetic species grown for biofuels such as maize, switchgrass, and sugarcane range from 5 Mg ha^–1^ year^–1^ to 26 Mg ha^–1^ year^–1^, and C_3_ photosynthetic species grown for biofuels such as oil palm, poplar, and willow produce between 2 Mg ha^–1^ year^–1^ and 14 Mg ha^–1^ year^–1^ (Somerville *et al.*, 2010). While these C_3_ and C_4_ crops are restricted to mesic environments, CAM crops such as *Agave* can grow on suboptimal soils in very dry conditions.

*Agave* varieties are now recognized as potential biofuel crops (Borland *et al.*, 2009; Davis *et al.*, 2010; Owen and Griffiths, 2013; Mielenz, 2015), but have been used in the past for beverages, food, fiber, medicines, shelter, and ornamentals (Garcia-Moya *et al*., 2011; Thakur *et al*., 2015). *Agave americana* is an obligate CAM species (Neales *et al*., 1968) that has recently been identified as having viable yields for biofuel production in arid conditions (Davis *et al.*, 2016). A defining characteristic of an obligate CAM species is nocturnal assimilation of CO_2_ by regulating stomatal opening during the night, and closing stomata during the day. This is opposite from the diurnal activities in C_3_ and C_4_ plants that are vulnerable to water loss during the hottest part of the day. The nocturnal stomatal opening is a trait that allows for greater WUE in CAM species (Ting, 1985).

Fixation of CO_2_ in an obligate CAM plant occurs in four phases that take place over a 24 h period (Dittrich *et al.*, 1973; Osmond, 1978; Owen and Griffiths, 2013). Briefly, stomata open at night to allow for nocturnal CO_2_ fixation, and organic acids accumulate (phase 1); sunlight becomes available while stomata are still open at dawn, causing a spike in CO_2_ assimilation (phase 2); stomata shut as the organic acids are decarboxylated, causing cells to become CO_2_ enriched while preventing water loss when sunlight is available (phase 3); and finally stomata open as the sun sets, causing another brief spike in carbon assimilation at dusk (phase 4). The overnight build up of organic acids in the vacuoles of CAM plants during phase 1 decreases the tissue pH, and the decarboxylation of organic acids for photosynthesis during phase 3 gradually elevates the tissue pH again during the day until phase 4. This change in tissue pH allows for the quantification of nocturnal CO_2_ fixation by phosphoenolpyruvate carboxylase (PEPC) via acid titrations of tissue samples collected at dusk and dawn (Ting, 1985; Osmond *et al.*, 1989; Silvera *et al.*, 2009).

Tissue acidity analysis has been used to determine the change in the productivity of CAM plants in response to environmental conditions. These findings can then be used to build a predictive environmental productivity index (EPI) model that is tested and calibrated with actual field results (Nobel, 1988, 1990). EPI models have been developed from measurements of titratable acidity in several CAM species, including *Agave lechuguilla* (Nobel and Quero, 1986), *Agave tequilana* (Nobel and Valenzuela, 1987), *Agave deserti* (Nobel and Hartsock, 1986*a*), *Agave salmiana* (Nobel and Meyer, 1985), and *Opuntia ficus-indica* (Nobel and Hartstock, 1984). A recent study by Owen *et al.* (2016) has also shown that the nocturnal acidification of *Agave* tissues is dependent upon leaf age, and distance from the leaf base. Titratable acidity is not, however, a direct measure of photosynthetic responses to light, and neither a light response nor an EPI has yet been resolved for *A. americana.*

The EPI model for a CAM species is typically developed using empirical data from laboratory studies that describe changes in the titratable acidity overnight or the 24 h gas exchange in response to changes in the soil water potential, photosynthetically active radiation (PAR), and temperature (Nobel and Hartsock, 1986*a*; Nobel, 1988). The effect of each of these three abiotic factors is tested individually by varying treatment levels of one factor while all other conditions are kept constant. This allows for the determination of the amount of water, PAR, and temperature that is required for optimum productivity, as well as the proportional change in productivity that can be expected in response to any deviations from optimum conditions. Separate indices are derived to describe the responses to water, PAR, and temperature, where an index of 1.00 corresponds to an optimum condition, and 0.00–0.99 is the proportional index for deviation from the optimum according to experimentally resolved relationships between production and environmental conditions. The product of the light, water, and temperature index values is equal to the EPI:

Light index×Water index×Temperature index=EPI

EPI models are useful tools for projecting the potential geographical ranges for CAM plant species (Nobel and Hartsock, 1986*a*; Garcia-Moya *et al.*, 2011), but no EPI models have previously been developed for *A. americana*. The goal of this study was to develop an EPI model for *A. americana* that can be used to predict the yield of this species in variable environmental conditions. From 2012 to 2016, *A. americana* was grown experimentally at the University of Arizona Maricopa Agricultural Center (Davis *et al.*, 2016). This was the first field experiment in which the yield of *A. americana* productivity was evaluated under different irrigation treatments. Here, we derive a model of productivity from the quantitative comparison of an empirically developed EPI. An EPI value was calculated for each month, and the sum of these monthly EPI values was compared with biomass yields measured in the field site in Maricopa, AZ.

Previous literature describes the response of gas exchange in *A. americana* to drought (Ehrler, 1983) and different temperatures (Neales, 1973; Nobel and Smith, 1983). However, the response of CO_2_ assimilation rates in *A. americana* to irradiance is still not well understood. In this study, we experimentally manipulated the light environment around *A. americana* plants to develop a light–response curve for this species. Gas exchange was measured continuously over 24 h after acclimation to each light level following methods previously used to study other CAM species (Nobel and Hartstock, 1983; Martin *et al.*, 1986; Cui and Nobel, 1994; Haslam *et al.*, 2003; Keller and Lüttge, 2005; Ceusters *et al.*, 2011, 2014).

The main objective of this study was to develop an EPI model that predicts *A. americana* biomass and energy yield in response to environmental conditions at a potential growing site. We first quantified, through experimental manipulation, the response of photosynthetic activity in *A. americana* to light and developed a light index that is compatible with the EPI model. We then quantified the response of *A. americana* production to water inputs using previously published data from the field site in Maricopa, AZ (Davis *et al.*, 2016), and developed a temperature index using previously published data sets that describe the response of *A. americana* production to temperature. The responses to light, water, and temperature were then converted to indices representing the proportional responses relative to the optimum for each condition. The resulting EPI model was parameterized for conditions in Maricopa, AZ, and the resulting indices for different field treatments were compared with actual biomass measurements. We also measured the energy content in *A. americana* plant tissues, and compared these data with EPI estimates to develop a predictive relationship for the theoretical energy yield of this species if cultivated as a bioenergy crop.

## Materials and methods

In December 2016, six 4-year-old *A. americana* individuals grown in a greenhouse at Ohio University were transplanted into large cylindrical pots (diameter=70 cm, height=19 cm) containing 47% Harvest organic potting soil (Harvest Power, Waltham, MA, USA) with an N-P-K of 10-5-5, 47% Country Side Accents potting soil (Grant County Mulch Inc., Arthur, WV, USA) with nutrient composition not quantified, and 6% Scotts turf builder fertilizer with an N-P-K of 32-0-4 (Scotts, Marysville, OH, USA). Pots were then placed in a growth chamber (Conviron; Winnipeg, Manitoba, Canada) with 12 h photoperiods and optimal 25/15 °C day/night temperatures (Neales, 1973; Nobel and Smith, 1983). The target PAR for each treatment was achieved using three mixed halogen/fluorescent light banks (Conviron) supplemented by three 1200 W LED grow lights (Roleadro COB; Shenzhen Houyi Lighting Co., Ltd, China) for higher light levels.

### Light treatments

The light environment was measured at mid-canopy using a LI-190 Quantum Sensor (Li-Cor, Lincoln, NE, USA). Six light treatments were applied as PAR photon flux densities (PPFDs) of ~100, 250, 500, 750, 1000, and 1250 µmol photons m^–2^ s^–1^ (as measured midcanopy in the plants). All six plants were given each light treatment simultaneously within the same growth chamber. The six different light treatments were applied in random order. All individuals were exposed to each light treatment for 10 d prior to gas exchange measurements to allow for enzymatic acclimation (Nobel, 1991). Plants were also watered to field capacity 48 h prior to gas exchange measurements to prevent physiological changes due to water limitations (Erhler, 1983).

At the end of the 10 d acclimation period with each new light treatment, 24 h gas exchange measurements were collected for all six individuals using three randomly assigned LI-COR 6400xt portable photosynthesis systems (Li-Cor Inc.). We selected the fourth leaf unfurled from the central spike for gas exchange measurements to avoid measuring gas exchange of leaves that were still developing (high canopy) or senescing (low canopy). For each 24 h measurement, the cuvette was clamped midway between the base and tip of the leaf and on tissue flanking the midrib. The cuvette was clamped using the thick gasket kit provided by Li-Cor^®^ to avoid potential leaks caused by thick leaves. The PAR at the specific surface measured on each leaf was recorded using a Li-Cor quantum sensor. Due to variation in light penetration of the plant canopy, PAR levels used in developing the light–response curves deviated from the target values described above; the measured light intercepted at the exact point of measurement was slightly different (±57 µmol photons m^–2^ s^–1^) from the average light environment in the plant canopy. During the 24 h measurement period, the leaf–atmosphere exchange of carbon dioxide was measured (µmol CO_2_ m^–2^ s^–1^) at 5 min intervals. The integrated net CO_2_ assimilated over each 24 h measurement was determined using Simpson’s rule (McKeeman, 1962) in the R studio statistical program.

### Light index

The photosynthetic response of *A. americana* to PAR was quantified by generating the best-fit quadratic equation that related the PAR (µmol photons m^–2^ s^–1^) treatments to the net moles of CO_2_ fixed over 24 h (MS Excel, Microsoft Corp). All replicated measurements were used to calculate this relationship. A light index for the EPI was then generated by converting the dependent light response variable to a proportional value that ranged from 0 to 1.00 at the maximum photosynthetic rate or the horizontal asymptote of the equation for the light response. An average daily PAR was calculated for each month of growth simulated for *A. americana*, and then used as a variable in the light index equation.

The monthly average solar irradiance (in Langley) recorded at the Arizona Meteorological Network (AZMET) automated weather station in Maricopa (https://ag.arizona.edu/azmet/az-data.htm) was converted to a monthly average daily PAR (in µmol photons m^–2^ s^–1^). The AZMET weather station recorded daily solar radiation in a unit that estimates energy distribution over an area (1 Langley=41 868 J m^–2^) (www.nist.gov), and 1 Langley is equivalent to 11.622 Wh of light in the visible spectrum (390–700 nm) per square meter. PAR is equal to the number of µmoles of photons hitting one square meter per second (µmol photons m^–2^ s^–1^) within the spectrum typically used by plants for photosynthesis (400–700 nm) (Enoch and Kimball, 1986).

Daily solar radiation was converted to an average PAR received in a particular day by first converting from Langley to Wh m^–2^, then dividing by the number of average daylight hours for that month and multiplying by the average global annual luminous efficacy value of 110 lumens W^–1^ (Littlefair, 1985). The resulting unit from these steps is lux (lumens m^–2^), which was converted into the average daily PAR using the relationship 1 klux=18 µmol photons m^–2^ s^–1^ of daylight (Li-Cor).

### Water index

*Agave americana* productivity from April 2012 to June 2015 under different irrigation treatments is described by Davis *et al.* (2016), and the resulting growth response to water in that study was used here to develop an equation describing the water response, and a water index for the EPI. The average monthly water input for each irrigation treatment was determined by dividing the total annual moisture received in each irrigation treatment by 12 (months). The relationship between water availability and biomass accumulation was resolved by regressing the annual average dry biomass against the average monthly water input.

### Temperature index

Previous results have shown no reductions in CO_2_ assimilation for *A. americana* due to high daytime temperatures until ~45 °C (maximum heat tolerance in *A. americana* is a staggering 63 °C) (Nobel and Smith, 1983). Monthly maximum temperatures did not exceed 41 °C at the field site for which the EPI was parameterized to compare with *A. americana* productivity. We therefore assumed that daytime temperatures have no effect on the temperature index, and would not have an effect in most potential growing locations. Night-time temperatures have the greatest effect on the net CO_2_ uptake of obligate CAM plant species (Nobel and Hartsock, 1978; Holtum and Winter, 2014). As such, the temperature index input values were determined using night-time temperatures, but adjusted to 2 °C above the monthly average minimum temperatures recorded at the AZMET meteorological station to correct for *A. americana* leaf temperatures that are 2 °C warmer on average than that of ambient night temperatures (Nobel, 1988; Davis, 2016).

The response of *A. americana* to shifts in night-time temperatures was determined from studies that quantified the response of titratable tissue acidity to experimentally decreased (Nobel and Smith, 1983) and increased (Neales, 1973) night-time temperatures while daytime temperatures were kept constant. The optimum night-time temperature reported in previous literature for *A. americana* is 15 °C, and productivity only ceases when night-time temperatures are ≤ –3 °C or ≥38 °C (Nobel and Smith 1983; Neales, 1972).

The quantitative relationship between the resulting index, proportional to the optimum, and the corresponding night-time temperature (°C) was used to calculate monthly temperature indices for the EPI using actual monthly average night-ime temperatures (+2 °C) at the Maricopa, AZ field site.

### EPI calculation

Monthly EPI values are equal to the product of water, temperature, and light index values. Monthly EPI values were calculated for the field site in Maricopa, AZ according to the monthly average PAR, monthly mean night-time temperatures in the experimental field plots, and the total monthly water inputs as measured in Davis *et al.* (2016). EPI values were calculated for plants grown in three of four irrigation treatments. The EPI of the fourth and highest irrigation treatment tested in the field study was excluded in comparisons with actual productivity measurements because of high mortality caused by a common native snout weevil, *Scyphophorus acupunctatus* (Davis *et al.*, 2016). The sum of the monthly EPI values for each treatment was compared with the average annual dry biomass yields of plants grown in the field for 2 years (measured in 2014) and plants grown in the field for 3 years (measured in 2015) (Davis *et al.*, 2016). The average total dry biomass of 2- and 3-year-old *A. americana* was regressed against the summed monthly EPI predictions over the life span of the plants to determine the quantitative relationship between the EPI estimates and biomass yields.

### Analysis of gross heat production

The energy content of *A. americana* plants was quantified so that a model could be derived that applied directly to projections of biomass energy if *A. americana* plants were developed as a bioenergy crop. Four plant samples were taken from each of the four irrigation treatments (*n*=16) in Maricopa, AZ, dried, weighed, and combusted in an isoperibol bomb calorimeter (Parr 6200EA, Parr Instrument Company, Moline, IL, USA) for gross heat analysis. In order to evaluate the gross heat of combustion (GH) for *A. americana* across all treatments, 2–3 g subsamples of biomass were placed into a custom-fabricated pelletizer and compressed using a 1 ton arbor press. Gross heat was then measured using the calorimeter, and a Parr 6510 water handling system (Parr Instrument Company) was used to maintain constant temperature. Benzoic acid (benzoic acid gross heat=6318 cal g^–1^) was used as a standard for calibration, and gross heat was adjusted for moisture content (GH_OD_) using the equation

$$GH_{OD}=GH_{sample}/\left(1-\frac{\%Moisture}{100}\right),$$

according to standard methods (Boundy *et al.*, 2011). A one-way ANOVA was performed using R Studio statistical software to determine if there was any significant difference in the GH produced for samples from different irrigation treatments. The experimentally resolved GH values were then plotted against the estimated EPI values calculated for the Maricopa, AZ field site to determine the strength of correlation between the estimated EPI values and the energy content of the plants.

## Results

### Light index

**Fig. 1.**
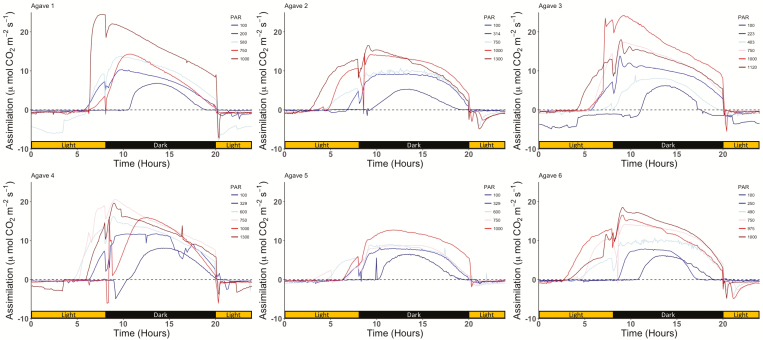
The 24 h rate and duration of CO_2_ assimilation after acclimation to varied levels of photosynthetically active radiation (PAR) (µmol photons m^–2^ s^–1^) in all six *A. americana* individuals tested. Black and yellow bars along the *x*-axis represent when lights were off and on, respectively, during the 24 h measurement period. The six *A. americana* individuals selected for this study were kept under a 12 h photoperiod of 25 °C/15 °C day/night temperatures.

As light intensity increased, both the duration and rate of CO_2_ assimilation by *A. americana* plants over the 24 h measurement periods increased (Fig. 1A–C). When acclimated and exposed to 100 µmol photons m^–2^ s^–1^, no carbon assimilation in CAM phases 2 or 4 was evident, and assimilation only occurred during the dark periods (phase 1) in *A. americana* individuals. All measurements at 250 µmol photons m^–2^ s^–1^ and above had distinct patterns in carbon assimilation during phase 4 in addition to phase 1. A pronounced period of CO_2_ assimilation in phase 2 was not observed in any of the light treatments. Occasional negative CO_2_ assimilation rates were measured during phase 2 and 3 at all light levels.

**Fig. 2.**
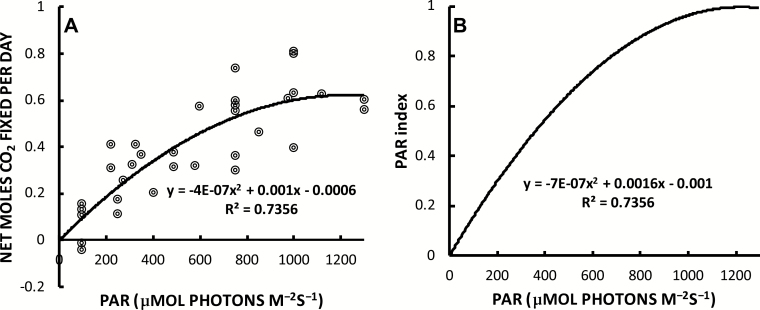
The relationship between the net moles of CO_2_ assimilated over 24 h versus the acclimated 12 h photoperiod PAR exposure (A) and the derived PAR index relationship (B) in *A. americana* with best-fit second-order polynomials (equations and *R*^2^ values are shown on the figure). The PAR intensity is equal to the exact PAR measured at the point on the leaf where gas exchange was measured.

The best-fit second-order polynomial describing the relationship between PAR intensity and carbon assimilation (24 h net moles) was equal to

$$y=–\text{4}\times \text{1}0^{–\text{7}}x^{\text{2}}+0.00\text{1}x–0.000\text{6}$$

where *x* is PAR in µmol photons m^–2^ s^–1^, and *y* is equal to the net moles of CO_2_ fixed in 24 h (Fig. 2A). Net 24 h CO_2_ assimilation peaks at 0.6244 mol d^–1^ with a light intensity of ~1250 µmol photons m^–2^ s^–1^. Two of the measurements made at 100 µmol photons m^–2^ s^–1^ had a net negative 24 h carbon assimilation rate (Fig. 2A). From this light response, a light index with values ranging from 0 to 1 was determined using the following equation:

$$\text{Light index}=–\text{7}\times \text{1}0^{–\text{7}}x^{\text{2}}+0.00\text{16}x–0.00\text{1}$$

where *x* is equal to daily PAR, averaged monthly in units of µmol photons m^–2^ s^–1^ (Fig. 2B).

### Water index

**Fig. 3.**
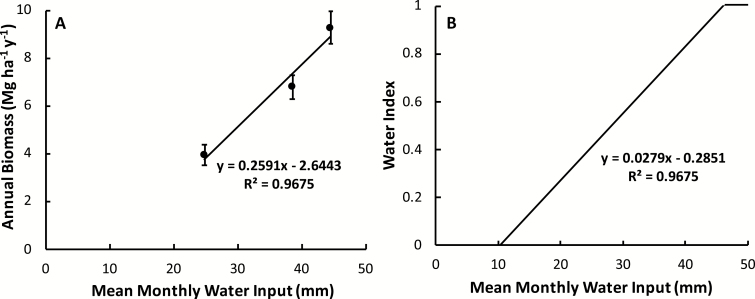
The annual gain in dry biomass in response to the mean monthly water input as described by Davis *et al.* (2016) (A), as well as the calculated water index relationship (B). Error bars represent the SE. Linear functions and *R*^2^ values are shown on their respective plots.

The relationship between the mean annual dry biomass gain and water inputs was represented by the linear equation

y=0.2591x–2.6443

where *x* is equal to the monthly mean water input (mm) and *y* is equivalent to the annual dry biomass gain (Mg ha^–1^ year^–1^). The maximum index value corresponded to a mean annual biomass of 9.27 Mg ha^–1^ year^–1^, as recorded in the experimental field trial with mean monthly annual inputs of 45 mm (Davis *et al*, 2016) (Fig. 3A).

The proportional biomass response to water inputs was determined by dividing the measured dry biomass by the maximum dry biomass measurement recorded. This percentage was then plotted against the total moisture inputs, including irrigation and precipitation (in mm), and the equation of the best-fit line through these points was used to calculate the water index:

Water index=0.0279x–0.2851

where *x* is equal to the monthly total moisture in mm (Fig. 3B).

 Because the best fit for the relationship between water and biomass was linear, a maximum index was established so that any monthly water inputs that would result in an index >1.00 will instead correspond to an index of 1.00 so that productivity does not exceed 100% of the maximum.

### Temperature index

**Fig. 4.**
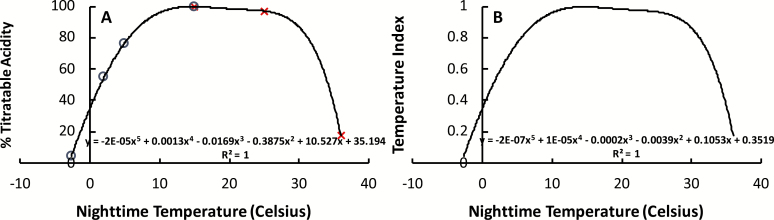
Change in nocturnal tissue acidity in *A. americana* tissues in response to variation in night-time temperatures as shown by the percentage of maximum recorded titratable acidity (A) and the calculated temperature index relationship (B). In (A), circles represent data collected by Nobel and Smith (1983), and the × symbols represent data from Neales (1972). The equations of the best-fit fifth-order polynomial and *R*^2^ values are displayed on the plots. (This figure is available in color at *JXB* online.)

The temperature index was calculated by combining the results from Nobel and Smith (1983) and Neales (1972) to describe a relationship between the fraction of maximum titratable tissue acidity and night-time temperature (°C) that was the best fit by the fifth-order polynomial

$$y=–\text{2}\times \text{1}0^{–\text{5}}x^{\text{5}}+0.00\text{13}x^{\text{4}}–0.0\text{169}x^{\text{3}}–0.\text{3875}x^{\text{2}}+\text{1}0.\text{527}x+\text{35}.\text{194}$$

where *x* is equal to the night-time temperature (°C) and *y* is the percentage of titratable acidity (Fig. 4A).

The temperature index was generated by dividing the titratable tissue acidity, which is a proxy for growth, by the maximum titratable acidity observed at optimum temperatures. This resulted in the following equation for the temperature index:

$$\begin{array}{c} \text{Temperature index}=–0.\text{2}\times \text{1}0^{–\text{7}}x^{\text{5}}+0.\text{13}\times \text{1}0^{–\text{4}}x^{\text{4}}–\text{1}.\text{66}\times \text{1}0^{–\text{4}}x^{\text{3}}\\ –\text{3}.\text{878}\times \text{1}0^{–\text{3}}x^{\text{2}}+0.\text{1}0\text{52}x+0.\text{352}0 \end{array}$$

where *x* is equal to the average monthly minimum night-time temperature in °C (Fig. 4B).

**Fig. 5.**
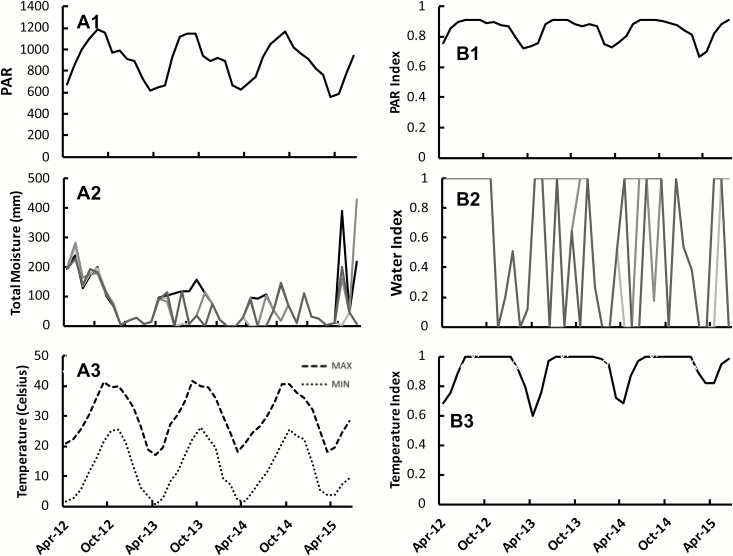
Monthly average PAR (A1), total moisture (A2), and max/min temperatures (A3) at field site in Maricopa, AZ; and mean monthly indexes for light (B1), water (B2), and temperature (B3) from April of 2012 to June of 2015. There were four different irrigation treatments at the AZ field site, generating four different monthly water index values (shown in A2, and B2 as gray scale lines where 780 mm=black, 530 mm=dark gray, 460 mm=gray, 300=light gray).

Water was most limiting to productivity in Maricopa, AZ, with an index value of 0.00 during the winter months (November–February) for all irrigation treatments after plants were established (Fig. 5B2). Light was the second most limiting (Fig. 5B3), and overall temperature was the least limiting of the three abiotic variables, with an index value of 1.00 during the summer months (May–August) (Fig. 5B1).

### EPI

**Fig. 6.**
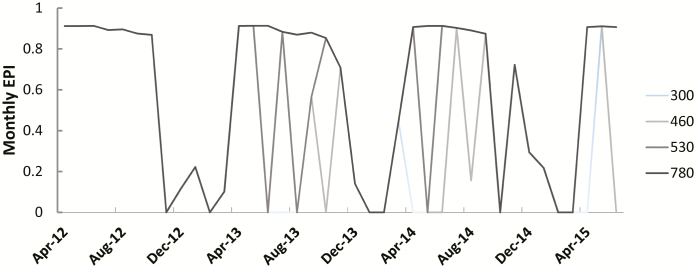
Calculated monthly EPI values for the 300, 460, 530, and 780 mm annual irrigation treatments in Maricopa, AZ from April 2012 to June 2015.

**Fig. 7.**
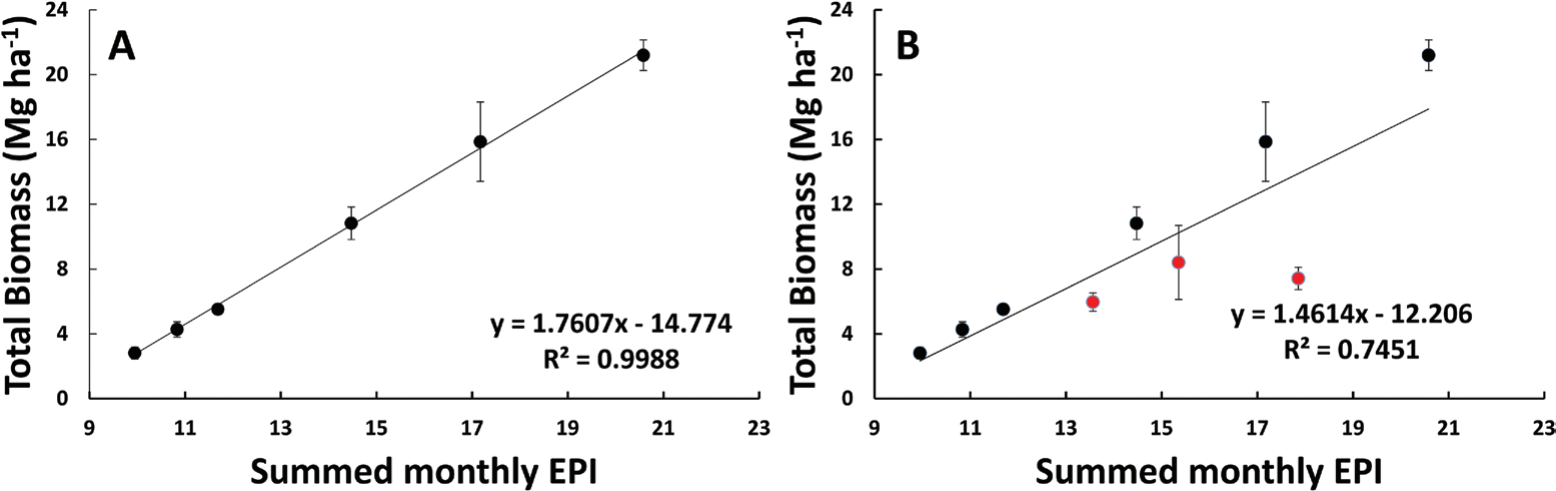
The total gain in biomass versus the summed monthly EPI estimates (from April of 2012 to June of 2015) for healthy *A. americana* individuals in Maricopa, AZ (A) as well as the total gain in biomass versus the summed monthly EPI estimates for all *A. americana* individuals including those killed by snout weevil (B). The best-fit linear equations and *R*^2^ values are shown on their respective plots. Error bars are representative of corresponding SE values. (This figure is available in color at *JXB* online.)

The monthly EPI values were summed over the lifetime of the plants (from April 2012 to June 2015), and resulting values for treatments with water inputs of 300, 460, and 530 mm in the field site were calculated as 14.47, 17.17, and 20.58, respectively. The monthly EPI values were equal to the product of light, water, and temperature index values for each month (Fig. 6). The summed monthly EPI values were strongly correlated (*R*^2^=0.9988) with the average total dry biomass of healthy 2- and 3-year-old *A. americana* individuals in AZ, resulting in the linear relationship

y=0.7607x–14.774

where *x* was equivalent to the summed monthly index values and *y* is equal to the average total dry biomass (Fig. 7A).

When biomass was calculated using plot averages that included plants affected by the pest *S. acupunctatus*, however, the correlation coefficient was reduced (*R*^2^=0.7451) (Fig. 7B). This is due to *S. acupunctatus* causing a reduction in the total average biomass that is not accounted for in the EPI model.

**Fig. 8.**
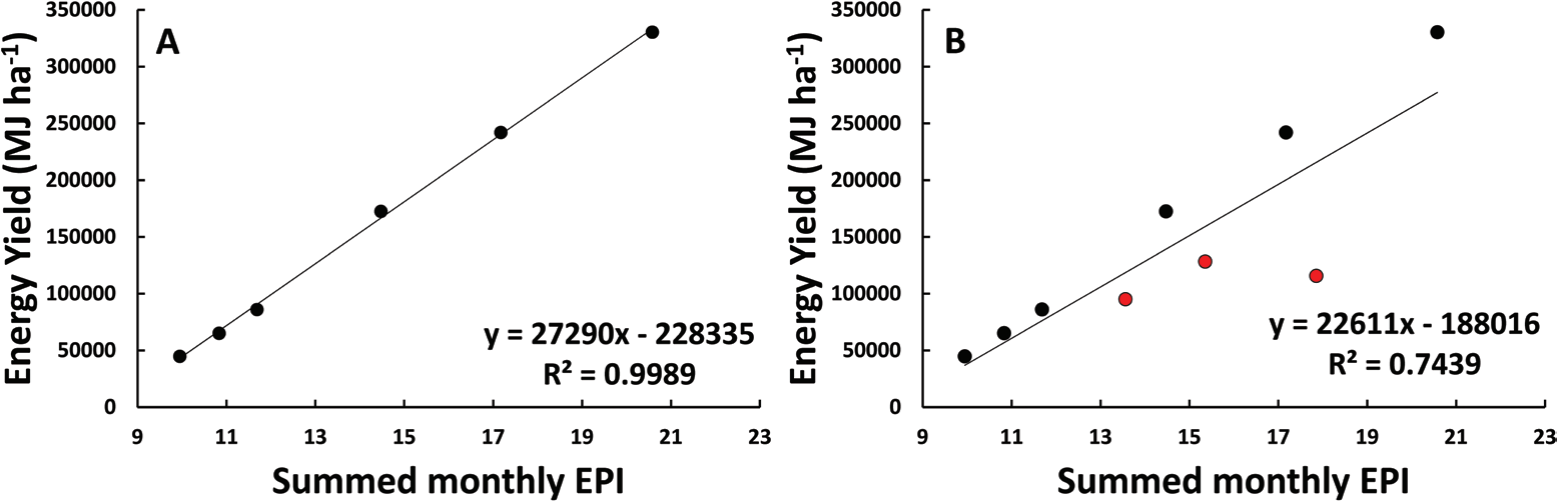
The combustible energy yield versus the summed monthly EPI estimates for healthy *A. americana* individuals in Maricopa, AZ (A) as well as the theoretical combustible energy yield versus the summed monthly EPI estimates for all *A. americana* individuals including those killed by the snout weevil (red circles) (B). The best-fit linear equations and *R*^2^ values are shown on their respective plots. Error bars are representative of corresponding SE values. (This figure is available in color at *JXB* online.)

A one-way ANOVA showed no significant difference in the energy content of plant tissues from plots with different irrigation treatments. The mean energy released from aboveground *A. americana* biomass was 15.44 MJ kg^–1^ (±0.42 MJ kg^–1^) across all treatments. The summed monthly EPI values were strongly correlated (*R*^2^=0.9989) with the energy yield (MJ ha^–1^) for healthy plants, and this relationship can be described by the linear equation

y=27 290x–228 335

where *x* equals the monthly summed EPI and *y* is equal to the combustible energy release (MJ ha^–1^) (Fig. 8A). As with biomass, this correlation is not as strong (*R*^2^=0.7439) when plot-level mortality from the pest *S. acupunctatus* is included in the measured comparison (Fig. 8B).

## Discussion

This study was the first to measure the photosynthetic response of *A. americana* to varied light levels, and this response was essential to characterize before an EPI model could be developed to predict potential productivity of this emerging crop species. Optimum light conditions resolved here for *A. americana* correspond to a higher light saturation point than that of other large agricultural CAM species, such as other *Agave* spp., *Opuntia* spp., and *Ananas* spp. (e.g. Nobel, 1988; X. Yang *et al.*, 2015). Other highly productive CAM species typically have a light saturation point near 30 mol photons m^–2^ d^–1^ (Nobel, 1988). The experimentally determined light saturation point of *A. americana* was found here to be 54 mol photons m^–2^ d^–1^, which is possibly because this species is adapted to the Sonoran Desert of northern Mexico and the Southwestern USA (Gentry, 1982), in a range with high average annual solar irradiance (https://www.nrel.gov/gis/solar.html).

The actual biomass accumulated by 2- and 3-year-old *A. americana* plants grown in Maricopa, AZ was highly correlated with EPI estimates for the site (Fig. 7), suggesting that the model developed here is a useful tool for projecting potential productivity of *A. americana* in areas where no yield data are available. Furthermore, the EPI relationship to the experimentally determined energy content of the plants can be used to estimate the energy yield of *A. americana* per unit of land area if grown as a bioenergy crop (Fig. 8).

The EPI developed for *A. americana* grown in Maricopa, AZ revealed, not surprisingly, that water was the most limiting of the three abiotic factors in question, especially during the winter months (Fig. 4, November–February) in which there were often little to no precipitation events. Even though productivity in *A. americana* was inhibited during drought, *Agave* species are capable of surviving over long periods with drought due to the presence of thick cuticular waxes (Deshmukh *et al.*, 2005) and succulent leaves. *Agave americana* also slightly retracts roots when soil water potentials become highly negative in order to sever the hydraulic soil–root connection, inhibiting water loss to dry soils (North and Nobel, 1997). Stomata remain shut during periods of drought to prevent any water loss from transpiration in a state of dormancy known as CAM-idling (Ting, 1987). These adaptations to arid environments allow CAM plants to survive in conditions that are intolerable for most C_3_ and C_4_ species.

While the yearly EPI predictions were strongly correlated with the average biomass accumulation in healthy *A. americana* individuals, EPI predictions cannot account for other limitations on biomass, such as the reduced biomass in response to a snout weevil infestation (Waring and Smith, 1986). Higher water inputs resulted in a higher percentage of plants infested by the snout weevil in the Maricopa, AZ field trial (Davis *et al.* 2016). The establishment and presence of *S. acupunctatus* is often difficult to diagnose in *A. americana*, as individuals remain in a vegetative state before showing symptoms (Waring and Smith, 1986; Kelly and Olsen, 2011; Davis *et al.*, 2016). Future efforts to produce crops of *A. americana* may depend on timing harvests according to *S. acupunctatus* outbreaks (Davis *et al.*, 2016), developing means to control *S. acupunctatus* infestation (Kelly and Olsen, 2011), or growing *A. americana* in areas where *S. acupunctatus* does not currently exist, such as Australia (Holtum *et al.*, 2011).

The light response calculated in this study was accomplished using 4-year-old plants, but responses to PAR may change with the age of *A. americana* plants (Nobel, 1986, 2003). As a species adapted to high light conditions, the striated cell walls of *A. americana* induce a relatively high level of light scattering and reflectance (Gausman, 1977). The photosynthetic response to sunlight in *A. americana* changes as the leaf area, leaf angles (Woodhouse *et al.*, 1980; de Cortázar and Nobel, 1986; Nobel, 2003), and spectral reflectance ( Christensen and Goudriaan, 1993; Peñuelas *et al.*, 1995) change with plant growth. For at least 10 years, *A. americana* grows continuously while producing clonal offshoots or ‘pups’ via ramets (Escobar-Guzman *et al.*, 2008). Following this vegetative period, the leaves begin to senesce, a single inflorescence bolts from the central spike, and the plant dies after producing seed. It is not well known how light responses change over the course of this life cycle, but an increase in surface area is expected to increase the total carbon gain in constant light conditions. The optimal light level per unit leaf area, as measured here, is not expected to have significant variation for the duration of the vegetative growth period. Future studies to understand how the canopy photosynthetic response changes due to the geometry of *A. americana* rosettes, leaf size, spectral reflectance, and angle as plants age could be used to calibrate the light index equation for all plant ages (Davis *et al.*, 2015).

Variation in the net moles of CO_2_ fixed over 24 h among individuals acclimated to the same light level was probably an effect of the differences between genotypes (Sultan, 2000), as the *A. americana* individuals measured in this study were not genetically identical clones, and varied in size. These plants represent a subsample of genetically variable individuals grown in Maricopa, AZ. The relationship defined here should be a reasonable approximation of the average photosynthetic response of *A. americana* in field conditions, but there is clearly potential for physiological crop improvement through genetic selection, breeding, or genetic modification.

The equation describing the water index for *A. americana* was derived by comparing the average annual biomass gain of plants in the Maricopa, AZ field site with the mean monthly water input per year. Since comparisons between the EPI estimates and actual growth in this study were made using biomass measurements from the same field site, the water index is still in need of validation in other field sites and/or through comparing EPI predictions with other *A. americana* growing operations. While research exists on how volumetric soil moisture content affects the transpiration rate in *A. americana* (Ehrler, 1983), an experiment measuring the response of nocturnal carbon uptake to soil water potentials that range from field capacity to permanent wilting point may improve model accuracy. Existing models that predict how desert soil water potentials are altered by precipitation and drought (Young and Nobel, 1986; Reynolds *et al.*, 2000, 2004) would allow for more precise predictions of productivity for *A. americana* in desert habitats of all soil types. This same method has been used for constructing the water index for several other *Agave* and *Opuntia* species (Nobel and Hartsock, 1984,1986*a*, *b*; Nobel and Meyer, 1985; Nobel and Quero, 1986; Nobel and Valenzuela, 1987; Nobel, 1988).

Similar to our observation, past studies determined that low and high night-time temperatures limit gas exchange more so than low and high daytime temperatures in xeric and semi-arid regions (Fig. 4) (Neales, 1973; Nobel and Hartsock, 1978; Nobel and Smith, 1983). Cold tolerance limits in *A. americana* have been experimentally determined to shift 1.8 °C for every 10 °C decrease in day/night growing condition temperatures, and the minimum cold tolerance (point of 50% cell death) is –7.4 °C (Nobel and Smith, 1983). The average monthly minimum temperatures in Maricopa never dropped below 0 °C (Fig. 5C3). *Agave americana* had negligible mortality rates in this field site due to low night-time temperatures (Davis *et al.*, 2016). High-temperature hardening in *A. americana* occurs with a 3.3 °C increase in the tolerance maximum for every 10 °C increase in day/night growing condition temperatures, and the maximum high temperature tolerance is 63.8 °C (Nobel and Smith, 1983). This maximum never occurred in the field site evaluated here (Fig. 5A3) and is unlikely to occur in most potential growing locations. When considering alternative sites for *A. americana* agriculture, low temperatures are more likely to have an effect on carbon assimilation and growth.

The combustible energy yield in *A. americana* of 15.44 MJ kg^–1^ was less than that of other large agricultural CAM plants such as *Agave tequilana* (17.50 ± 0.09 MJ kg^–1^), and slightly less than that of *O. ficus-indica* (16.95 ± 0.04 MJ kg^–1^) (L. Yang *et al.*, 2015). However, while the methods of this study were similar, L. Yang *et al*. (2015) placed tissue samples in a hydraulic press before combustion, which removed the juice fraction. Doing the same for *A. americana* may slightly increase the combustible energy yield per unit mass. The EPI model developed here can be used to estimate and compare the amount of energy that could be produced at different localities (Fig. 8). Similar studies have been reported in previous literature using EPI models for *A. fourcroydes*, *A. salmiana*, *A. tequiliana*, and *O. ficus-indica* (Owen and Griffiths, 2014).

Although the goal of this study was to use photosynthetic responses of light, water, and temperature to parameterize a productivity model, adding a nutrient index parameter may also improve estimates of productivity for certain regions (Nobel, 1988, 1989). Adding a nutrient index into the EPI model for *A. americana* would probably have little effect on the EPI values calculated for the Maricopa field site, as all irrigation treatment groups were fertilized on an annual basis and nutrients were not limiting (Davis *et al.*, 2016). Still, the photosynthetic response of *A. americana* to variation in soil nutrient content may prove useful in defining the minimum amount of nitrogen fertilizer necessary for optimum carbon fixation.

In facultative CAM plants, variation in N application has been shown to induce or suppress CAM expression (Santos and Salema, 1991; Winter and Holtum, 2011). The specific response varies among species, as does the preference for nitrate versus ammonium. For example, the facultative CAM plant *Kalanchoe blossfeldiana* has been shown to shift into full CAM when supplied solely with nitrate (Ota, 1988; Ota *et al.*, 1988), while the facultative CAM species *Guzmania monostachia* has an increase in carbon assimilation by CAM photosynthesis when grown in ammonium (Pereira *et al.*, 2018). Variations in available phosphorus and potassium appear to have little effect on the induction of CAM (Rodrigues *et al.*, 2014), and do not limit growth in the majority of desert-adapted *Agave* and *Opuntia* spp. below 60 µg g^–1^ of phosphorus, and 250 µg g^–1^ of potassium in soil (Nobel, 1989).

CAM plants may have a higher nitrogen-use efficiency (NUE) than that of C_3_ plant species (Griffiths, 1989; Raven and Spicer, 1996), the hypothesis being that there is a reduced demand for Rubisco in CAM plants, which can make up 50% or more of the total soluble leaf protein (Lüttge, 2004). Such has been shown to be the case in C_4_ plant species (Monson, 1989; Marschner and Marschner, 2012). However, past comparisons of NUE in succulent CAM and C_3_ plant species suggest that results vary greatly with age and environmental conditions (Lüttge, 2004; Rodrigues *et al.*, 2014). Studies on *Kalanchoe pinnata* have shown that the investment in Rubisco significantly decreases as tissues mature and transition from C_3_ to obligate CAM photosynthesis with only a moderate increase in PEPC content (Winter *et al.*, 1982). Experiments measuring biomass accumulation with different nitrogen treatments in the CAM plants *K. daigremontiana* and *K. tubiflora* showed that biomass is drastically reduced when nitrogen is limiting compared with C_3_ species (Widmann *et al.*, 1990). CAM in semi-aquatic *Litterolla uniflorma* was found not to contribute to an increase in NUE (Baattrup-Pedersen and Madsen, 1999). Likewise, a study at Barro Colorado Island, Panama showed that the presence of CAM in epiphytes did not coincide with higher long-term NUE than that of sympatric C_3_ epiphytes (Zotz and Winter, 1994). More work is needed to understand NUE efficiency and growth responses of *A. americana* to changes in edaphic conditions.

A simple environmental productivity model developed for *A. americana* is a useful starting point for predicting potential yields in semi-arid and xeric regions around the world. The EPI model will be valuable for understanding geographic ranges favorable for *A. americana* as climate changes in the near future. While the current EPI model assumes that physiological limitations are dominated by light, temperature, and water, other indexes can be added to describe abiotic (e.g. soil nutrient content) or biotic (e.g. herbivory) variables that affect productivity as more information about *A. americana* stressors becomes available.
